# Dr. Jules Constantin

**Published:** 1913-01

**Authors:** 


Dr. Jules Constantin, formerly assistant to Dr. Doyen of Paris, was killed by bullets through the breast, while manipulating an aeroplane in the Bulgarian army service. After receiving the fatal wound, he made a return to the Bulgarian camp and a safe landing but was found dead at the helm, immediately afterward.

